# Zinc as an adjunct therapy in the management of severe pneumonia among Gambian children: randomized controlled trial

**DOI:** 10.7189/jogh.08.010418

**Published:** 2018-06

**Authors:** Stephen Howie, Christian Bottomley, Osaretin Chimah, Readon Ideh, Bernard Ebruke, Uduak Okomo, Charles Onyeama, Simon Donkor, Onike Rodrigues, Mary Tapgun, Marie Janneh, Claire Oluwalana, Bankole Kuti, Godwin Enwere, Pamela Esangbedo, Conor Doherty, Grant Mackenzie, Brian Greenwood, Tumani Corrah, Andrew Prentice, Richard Adegbola, Syed Zaman

**Affiliations:** 1 Medical Research Council Unit The Gambia, Fajara, Gambia; 2 Department of Paediatrics: Child and Youth Health, University of Auckland, Auckland, New Zealand; 3 Centre for International Health, University of Otago, Dunedin, New Zealand; 4 London School of Hygiene and Tropical Medicine, London, UK; 5 Edward Frances Small Teaching Hospital, Banjul, Gambia; 6 Murdoch Childrens Research Institute, Melbourne, Australia; 7 Liverpool School of Tropical Medicine, Liverpool, UK

## Abstract

**Background:**

The benefit of zinc as an adjunct therapy for severe pneumonia is not established. We assessed the benefit of adjunct zinc therapy for severe pneumonia in children and determined whether the study children were zinc deficient.

**Methods:**

This was a randomized, parallel group, double-blind, placebo-controlled trial with an allocation ratio of 1:1 conducted in children with severe pneumonia to evaluate the efficacy of daily zinc as an adjunct treatment in preventing ‘treatment failure’ (presence of any sign of severe pneumonia) on day-5 and day-10 and in reducing the time to resolution of signs of severe pneumonia. Six hundred and four children 2-59 months of age presenting with severe pneumonia at six urban and rural health care facilities in The Gambia were individually randomised to receive placebo (n = 301) or zinc (n = 303) for seven days. To determine if the study children were zinc deficient, supplementation was continued in a randomly selected subgroup of 121 children from each arm for six months post-enrolment, and height-gain, nutritional status, plasma zinc concentrations, and immune competence were compared.

**Results:**

Percentage of treatment failure were similar in placebo and zinc arms both on day 5 (14.0% vs 14.1%) and day 10 (5.2% vs 5.9%). The time to recovery from lower chest wall indrawing and sternal retraction was longer in the placebo compared to zinc arm (24.4 vs 23.0 hours; *P* = 0.011 and 18.7 vs 11.0 hours; *P* = 0.006 respectively). The time to resolution for all respiratory symptoms of severity was not significantly different between placebo and zinc arms (42.3 vs 30.9 hours respectively; *P* = 0.242). In the six months follow-up sub-group, there was no significant difference in height gain, height-for-age and weight-for-height Z-scores, mid upper arm circumference, plasma zinc concentrations, and anergy at six months post-enrolment.

**Conclusions:**

In this population, zinc given as an adjunct treatment for severe pneumonia showed no benefit in treatment failure rates, or clinically important benefit in time to recovery from respiratory symptoms and showed marginal benefit in rapidity of resolution of some signs of severity. This finding does not support routine use of zinc as an adjunct treatment in severe pneumonia in generally zinc replete children.

**Trial registration:**

ISRCTN33548493.

Pneumonia is the leading cause of death in children worldwide, accounting for 17% of almost 6 million deaths in children under 5 years of age in 2015, most of these occurring in sub-Saharan Africa [1,2]. In Gambia, pneumonia is a leading cause of death in young children [3,4].

To achieve the Global Action Plan for the Prevention and Control of Pneumonia (GAPPD) goal of reducing mortality from pneumonia in children under 5 years of age to fewer than 3 per 1000 live births by 2025 innovative and effective interventions are required [5]. One such potential intervention is therapeutic zinc.

Zinc deficiency impairs growth and may contribute to child death due to pneumonia. However, the beneficial impact of zinc supplementation on respiratory infection is not clear. A meta-analysis of preventive zinc supplementation trials showed a significant reduction in the incidence and prevalence of pneumonia among children of 2 to 59 months of age in Bangladesh, India, Peru, and South Africa [6]. However, a meta-analysis of trials of therapeutic zinc supplementation as an adjunct to antibiotic for treatment of severe and non-severe pneumonia in Bangladesh, Nepal and India did not show any benefit [7], although an earlier study in Bangladesh [8] showed beneficial impact.

Zinc deficiency is associated with diets high in phytate and fibre and low in protein. Because most of the dietary energy intake in Gambian children is derived from cereals and ground nuts, and intake of animal products is extremely low, the Gambian diet places children at risk of zinc deficiency [9]. The best method of confirming zinc deficiency in a population is to show a positive response to supplementation through well-designed randomized controlled trials [10]. Therefore, we conducted this trial to assess the benefit of short-term adjunct zinc therapy for severe pneumonia in children and also to determine whether the study children had clinically measureable zinc deficiency by assessing the impact of continued long term supplementation of zinc on growth and immune competence after recovery from severe pneumonia.

## Methods

### Study setting

**Figure 1:**
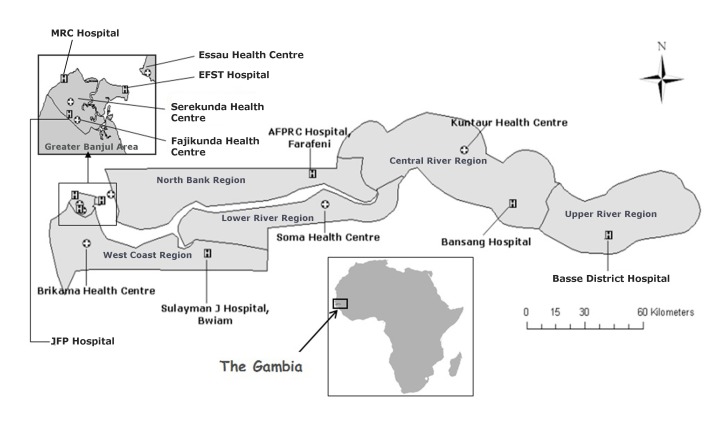
Map of Gambia showing study sites: Greater Banjul area (periurban site) and Upper River Region (rural site).

The Gambia is a small West African country of 1.8 million population with an HIV infection prevalence of less than 2% [11]. A randomized, parallel group, double-blind, placebo-controlled trial with an allocation ratio of 1:1 was conducted in children with severe pneumonia to evaluate the efficacy of daily zinc as an adjunct treatment in preventing ‘treatment failure’ and reducing the time to resolution of signs of severe pneumonia. The trial was undertaken at a peri-urban (the Greater Banjul area) and a rural (the Upper River Region) setting (**Figure 1**). Participants were recruited from the MRC hospital, EFST Hospital, Basse District Hospital, and the major health centres at Fajikunda, Serekunda, and Brikama.

### Procedures

#### Randomisation and blinding

An independent statistician allocated treatment in 604 children to one of the two arms by individual randomisation. Unique identification numbers of study children were randomly allocated to placebo or zinc using block randomisation (block size = 10). Separate randomisation lists were generated for each health facility, with separate lists for children aged 2-12 months and 12-59 months, using Stata version 12.0. A randomly selected list of 242 children (121 in each arm) was generated at the outset for the subset of children enrolled for the study of 6 months zinc supplementation post-enrolment. The randomisation lists with allocation were kept by the Data Safety Monitoring Board. The study code was broken after all data were entered and initial blinded analysis had been performed.

Each tablet contained placebo or 10 mg elemental zinc as zinc sulphate (NUTRISET-B.P. 35-76770, Malaunay, France). The tablets were water-dispersible, identical in appearance, smell and taste, and were supplied in blister packs of 7 or 14 tablets. The blister packs were identical in appearance except for the batch numbers (which identified whether the pack contained placebo or zinc) and the expiry date printed at the edge. Individual randomisation packs numbered to correspond to the randomisation lists were prepared and the printing at the edge of the blister pack was cut off to ensure blinding.

We decided to give fixed doses of 10 mg elemental zinc daily for infants and 20 mg daily for children between 12 and 59 months of age as the vast majority of published zinc supplementation trials have used these doses without any adverse effect. In this trial zinc was administered for 7 days, as the response to zinc repletion is extremely rapid because of small body zinc pool and in an earlier trial beneficial impact on recovery from severe pneumonia was observed within 7 days [8]. For the subgroup of 242 children included for the six month follow-up study, supplementation was continued with the same dosage for a total of 6 months post-enrolment.

#### Enrolment

All children who presented with a history of cough or difficulty in breathing at the outpatient departments were screened by nurses for eligibility. Substantial training of study nurses on WHO criteria for classification and management of children with cough or difficulty in breathing was conducted and quality control of counting of respiratory rates and recognition of severe pneumonia instituted. Cases were children aged 2-59 months with severe pneumonia defined clinically by modified WHO criteria [12] (cough or difficulty in breathing plus any of the following: lower chest wall indrawing (LCWI), nasal flaring, or an oxygen saturation of <90% on pulse oximetry, the latter defining very severe pneumonia). Children with a cough of ≥2 weeks duration, with known congenital or metabolic abnormality, convulsion or unconsciousness, severe malnutrition (defined as weight-for-height z-score<-3 or oedema of both feet (WHO) [12], clinically suspected systemic infection other than pneumonia, confirmed wheeze at recruitment, severe anaemia (PCV<18%, or Hb <6g/dL), or living outside the study area were excluded from the study.

A study paediatrician confirmed eligibility. The parents or the main caregivers were informed about the details of the trial, invited to allow their child to participate, and written informed consent obtained. The child was randomised to a treatment arm by the study paediatrician using the randomisation lists. Clinical findings were recorded on pre-tested standardised clinical record forms.

#### Administration of zinc and placebo

Placebo or zinc was administered by study nurses within one hour of administration of antibiotics. Subsequent doses were administered once daily before the first morning feed. Each child received supplementation for 7 days. A randomly selected subgroup of 242 children continued receiving study medication at home weekly for six months in total. During the weekly visits the health worker collected the previous supplement packaging and recorded the amount of left over tablets. A standard multivitamin supplement was given to all children during hospitalisation while no additional mineral supplements were given.

#### Anthropometry

WHO guidelines were followed for measuring weight and length [13]. Clinical staff and field workers were trained and standardised in the measurement of weight and height before starting the trial and the process of standardisation was repeated periodically throughout the trial. Length/height was measured using length boards/stadiometers (ShorrBoard^R^, Maryland, USA) with a precision of 0.1cm and weight was measured using electronic weighing scales (Digital Baby Scale, Model 727, Seca, Hamburg, Germany) with a precision of 10 grams. Weight was measured daily during hospitalisation.

#### Inpatient clinical care

All participants were given standardised clinical care as inpatients based on WHO guidelines [12] and standard operating procedures. This included antibiotic therapy for severe pneumonia and all supportive care. Those with severe pneumonia were reviewed a minimum of once daily by a medical doctor and 6 hourly by a nurse; for those with very severe pneumonia a minimum of twice daily medical review and 3 hourly nursing review was undertaken. Oxygen saturation was measured using standard commercially available pulse oximeters. For patients receiving oxygen, the oxygen cannula was withdrawn for five minutes and respiratory rate and signs of severe pneumonia and oxygen saturation were measured. Axillary temperature was measured using a digital thermometer. The study paediatricians confirmed treatment failure (defined below) and antibiotic therapy was changed according to WHO guidelines. All cases of treatment failure were closely observed until resolution of the signs. Children were discharged when all of the following conditions were met for a minimum of 24 consecutive hours: signs of severe or very severe pneumonia were no longer present; fast breathing had resolved; axillary temperature <37.5°C; and no other contraindication to discharge was present.

Children discharged before day-5 were visited by a health worker at day-5 and day-10, and children discharged between day-5 and day-10 were visited on day-10.

#### Laboratory tests

Two thick blood films for malaria were examined at day-0 and on any day during follow-up in the sub-group with 6 months follow-up if there was a temperature of >37.5°C. On enrolment full blood count and blood cultures were performed. A chest x-ray was performed on all participants as soon as possible after enrolment.

Plasma zinc, ferritin, and soluble transferrin receptor (sTfR) were measured for the sub-group of children at enrolment and at 6 months post-enrolment at the MRC Human Nutrition Centre in Cambridge. Zinc was measured on Siemens Dimension Xpand using a reagent kit manufactured by Audit Diagnostics (Carrigtwohill, Co. Cork, Republic of Ireland). Ferritin was measured on Siemens Dimension Xpand using Siemens reagent FERR. sTfR was measured by ELISA using the Ramco kit, semiautomated on the BEST sample processor (Launch Diagnostics). All assays were conducted in accordance with standard operating procedures and ISO 9001:2008, including quality control samples alongside study samples and subscribing to national external quality assessment schemes where available. Samples for these tests were collected using trace element free needle, syringe and tubes. Soluble transferrin receptor - ferritin (sTfR-F) index was calculated as the ratio of sTfR to log ferritin values. The sTfR-F index gives an estimate of total body iron and is considered a more reliable index than plasma ferritin and other conventional laboratory iron parameters in diagnosing iron deficiently anaemia (IDA) and, in non-inflamed individuals, is also as reliable as bone marrow aspirate in detecting iron stores in patients with IDA [14].

#### Cell-mediated immunity (CMI) skin test

A CMI skin test was conducted at 6 months post-enrolment in the sub-group receiving six months zinc supplementation. The kit (Merieux Multitest, Insitut Merieux, Lyon, France) comprised a sterile disposable plastic multipuncture applicator with eight heads preloaded with standardized doses of seven antigens (Tetanus, Diphtheria, Streptococcus, tuberculosis, Candida, Trychophyton, and Proteus) in glycerine solution and a glycerine-negative control that were applied to the volar surface of the right arm of each child. The diameter of each area of induration was measured by specially-trained nurses 48 hours after administration using the pen method [15]. A reaction was considered positive if the average of the maximum horizontal and vertical induration was at least 2 mm.

### Sample size

The original sample size calculation for the impact of zinc supplementation on severe pneumonia outcome was based on data from an RCT conducted in a non-stunted but generally zinc-deficient population in Bangladesh [8]. In this study failure rates of 3% in the supplemented group and 11% in the unsupplemented group were observed in children without wheezing. We expected failure rates to be 30% lower in non-stunted Gambian children than Bangladeshi children (i.e 7.5% in the placebo group and 1.8% in the zinc group). Based on this assumption and allowing for 20% dropout, we aimed to recruit 300 children per arm to have 80% power to detect a difference between the arms at an alpha level of 0.05.

The sample size for length gain following zinc supplementation for 6 months post-discharge was based on Ethiopian data [16] in stunted children where the length gain after 6 months was 7.0 cm (SD±7.37) in the zinc-supplemented group and 2.8 cm (SD±6.03) in the placebo group. We anticipated a length gain of 5.5 cm in the supplemented group and 2.8 cm in the placebo group and similar standard deviations. Allowing for 20% dropout, we therefore aimed to recruit 120 in each arm to have 80% power to detect a difference between the study arms at an alpha level of 0.05.

### Data analysis

Baseline characteristics of the placebo and zinc arms were compared to check for any imbalance in prognostic factors. We undertook intention-to-treat analysis.

#### Short term endpoints

The primary endpoint of the trial was treatment failure on day-5 and day-10. Treatment failure was defined as the presence of cough or difficulty in breathing with at least one of the following signs: LCWI or nasal flaring or hypoxaemia (oxygen saturation <90%). The secondary outcomes were time to resolution of hypoxaemia, LCWI, nasal flaring, grunting, head nodding, sternal retraction, central cyanosis, fast-breathing (≥50 per min in children 2-11 months and ≥40 per min in children 12-59 months), all three main signs of severity (LCWI, nasal flaring, hypoxaemia), and time to discharge.

The proportion of children who failed to respond to treatment by day-5 was measured for the placebo and zinc groups. Children who died before day-5 were also classed as treatment failures, while children who were discharged before day-5 and lost to follow up (ie, not seen by a health worker at day-5) were excluded from the analysis. A logistic regression model was used to adjust for baseline imbalance in age, sex, height-for-age (HAZ) and weight-for-height (WHZ) Z-scores, and pneumonia severity. The HAZ and WHZ were calculated using the WHO standards as the reference distribution [17]. Treatment failure at day-10 was analysed similarly.

The Kaplan-Meier method was used to estimate the median time to resolution for each of the symptoms of severe and very severe pneumonia (hypoxaemia, LCWI, nasal flaring, grunting, sternal retraction, head nodding, central cyanosis, and fast-breathing) among those individuals who had the symptoms at baseline. In addition it was used to estimate the median time to the resolution of all signs of severity. For each symptom, we compared the placebo and zinc groups by fitting a Weibull accelerated failure time (AFT) model to the data. The regression parameter from this model - the ratio of median resolution times was used to quantify the effect of treatment on time to resolution and adjusted estimates were obtained by including the covariates listed above for the primary outcome in the model. For all time to event analyses (ie, Kaplan-Meier estimates and AFT models) children were censored at date of death, date of discharge or day-10 if the symptom(s) had not resolved at this point.

#### Long term endpoints

Rates of growth from enrolment to six months later were estimated separately for each child using ordinary least squares regression. Average rates of growth were presented separately by trial arm and the crude difference calculated. Linear regression was used to estimate the difference in growth rate (cm per month) between the trial arms adjusted for baseline age, sex, WHZ, HAZ, pneumonia severity, and zinc concentration.

Additionally we compared between trial arms mean height, HAZ, WHZ, plasma zinc, ferritin, sTfR concentrations, and sTfR-F index, CRP, the proportion of children who were anergic, and the proportion malnourished (using Mid Upper Arm Circumference) . Linear regression was used to estimate adjusted differences, except for anergy and malnutrition where crude odds ratios were used to compare trial arms since these were recorded as binary variables and there were too few children with these outcomes to use regression models. The linear regression models included covariates as listed above for the analysis of growth rate.

Changes in plasma zinc, ferritin, sTfR, sTfR-F, and CRP concentrations between baseline and six months were tested using Wilcoxon matched-pairs signed-ranks test.

### Ethics approval

The study was approved by the Gambian Government-Medical Research Council Joint Ethics Committee (SCC/EC1062). The trial was registered in the ISRCTN registry at www.isrctn.com as ISRCTN33548493, and run according to Good Clinical Practice guidelines.

## RESULTS

**Figure 2:**
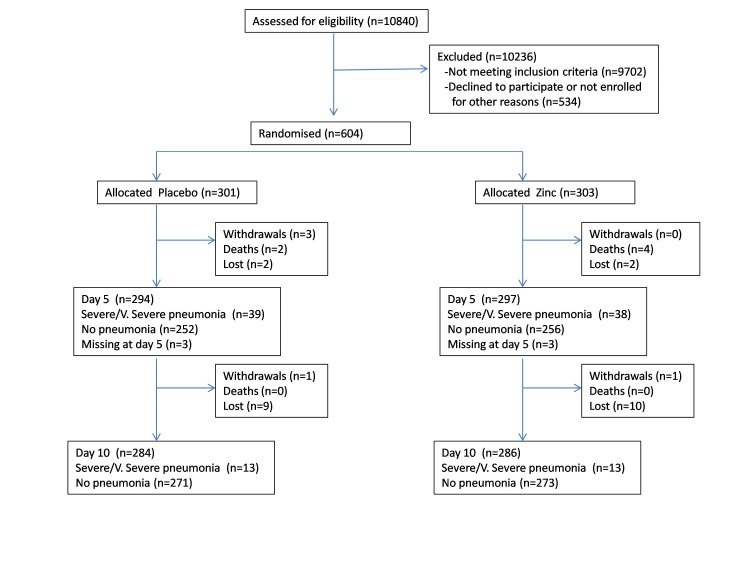
Profile of entry of patients into the trial (short term).

**Figure 3:**
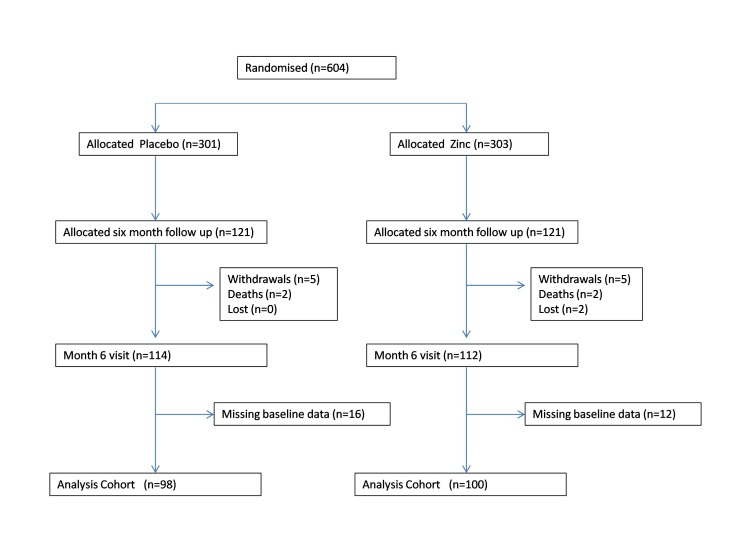
Profile of six months follow up (long term).

We recruited children between 28 November 2005 and 31 January 2011; follow up ended on 11 July 2011. 10 840 children were screened for eligibility, of which 604 were eligible and enrolled in the trial (**Figure 2**). 121 children in each arm were allocated for 6 months continued zinc supplementation for assessing the long-term impact of daily zinc supplementation in this population with 98 children in the placebo arm and 100 children in the zinc arm available for analysis at the end of the 6 months (**Figure 3**).

The characteristics of children at enrolment were similar in placebo and zinc arms (**Table 1**). Median age at enrolment in both arms was 13.0 months. 7.0% in the placebo and 4.0% in the zinc arm were severely stunted, and 8.4% in the placebo and 6.6% in the zinc arm were severely wasted, and 14.0% in the placebo arm and 12.2% in the zinc arm had hypoxaemia.

**Table 1:** Baseline characteristics of children with severe pneumonia enrolled in the trial

Characteristics		Placebo	Zinc
Gender	Male	165 (54.8)	163 (53.8)
	Female	136 (45.2)	140 (46.2)
Age (months)	2-5	56 (18.6)	66 (21.8)
	6-11	76 (25.2)	66 (21.8)
	12-23	93 (30.9)	100 (33.0)
	24-35	51 (16.9)	54 (17.8)
	36-59	25 (8.3)	16 (5.3)
	60+	0 (0.0)	1 (0.3)
Median (IQR)		13.0 (7.0, 24.0)	13.0 (6.0, 23.0)
Height-for-age z-score	≤-3.00	21 (7.0)	12 (4.0)
	-2.99 to -2.00	25 (8.4)	28 (9.3)
	-1.99 to -1.00	68 (22.8)	69 (22.8)
	>-1.00	184 (61.7)	193 (63.9)
	Median (IQR)	-0.7 (-1.6, 0.1)	-0.6 (-1.4, 0.3)
Weight-for-height Z-score	≤-3.00	25 (8.4)	20 (6.6)
	-2.99 to -2.00	75 (25.2)	52 (17.2)
	-1.99 to -1.00	97 (32.6)	110 (36.4)
	>-1.00	101 (33.9)	120 (39.7)
	Median (IQR)	-1.4 (-2.3,-0.8)	-1.3 (-2.0, -0.6)
Oxygen saturation (%)	≤89	42 (14.0)	37 (12.2)
	90-93	54 (18.1)	58 (19.1)
	≥100	203 (67.9)	208 (68.6)
	Median (IQR)	95.0 (93.0, 97.0)	95.0 (92.0, 97.0)
Respiratory rate	35-39	2 (0.7)	1 (0.3)
(breaths per minute)	40-49	25 (8.4)	32 (10.6)
	50-128	272 (91.0)	270 (89.1)
	Median (IQR)	64.0 (58.0, 72.0)	63.0 (54.0, 72.0)
Nasal flaring	No	54 (18.1)	60 (19.8)
	Yes	245 (81.9)	243 (80.2)
Lower chest wall indrawing	No	41 (13.7)	46 (15.2)
	Yes	258 (86.3)	257 (84.8)
Plasma zinc conc.(µmol/L)	Median (IQR)	(n = 104) 14.0(7.5, 23.7)	(n = 108) 11.3 (7.6, 19.4)
Plasma ferritin conc.(ng/ml)	Median (IQR)	(n = 108) 90.5(35.5, 165.5)	(n = 109) 104.0 (36.0, 198.0)
Soluble transferrin receptor conc. (µg/ml)	Median (IQR)	(n = 119) 7.4(5.3, 9.5)	(n = 115) 6.7 (4.9, 8.6)
Soluble transferrin- receptor-ferritin index (sTfR-F index)	Median (IQR)	(n = 108) 3.7(2.5, 5.8)	(n = 108) 3.3 (2.3, 5.3)
Plasma C-Reactive Protein (mg/L)	Median (IQR)	(n = 107) 13.5(0.6, 86.1)	(n = 107) 19.1 (0.6, 186.0)

IQR – interquartile range

There was no significant difference in the proportion of treatment failure on day-5 (14.0% vs 14.1%) or on day-10 (5.2% vs 5.9%) in placebo and zinc arm respectively (**Table 2**). The time to recovery from LCWI and sternal retraction was longer in the placebo compared to zinc arm (24.4 vs 23.0 hours; *P* = 0.011 and 18.7 vs 11.0 hours; *P* = 0.006 respectively). There was no significant difference in time to recovery for other symptoms measured.

**Table 2:** Treatment outcomes from 7 days of either placebo or zinc

Outcomes	Placebo n/N (%)	Zinc n/N (%)	OR (95% CI)*	*P*-value
**Treatment failure:**				
Day-5	41/293 (14.0)	42/298(14.1)	1.08(0.65,1.80)	0.773
Day-10	15/286 (5.2)	17/290(5.9)	1.18(0.54,2.60)	0.682
				
Median (IQR) time to resolution (hours)	**Placebo median (N)†**	**Zinc median (N)†**	**Ratio (95% CI)†**	***P*-value**
Oxygen saturation <90%	7.2 (42)	5.9 (36)	1.33 (0.82,2.14)	0.251
Lower chest wall indrawing	24.4 (258)	23.0 (257)	0.84 (0.74,0.96)	0.011
Nasal flaring	13.4 (245)	13.2 (243)	0.96 (0.83,1.11)	0.574
Grunting	4.9 (109)	5.3 (81)	1.11 (0.85,1.44)	0.448
Head nodding	4.0 (25)	3.3 (31)	0.71 (0.39,1.31)	0.274
Sternal retraction	18.7 (21)	11.0 (23)	0.51 (0.31,0.82)	0.006
Central cyanosis	1.4 (12)	1.8 (12)	0.60 (0.31,1.15)	0.121
Respiratory rate ≥50 per min	21.0 (272)	17.9 (270)	0.89 (0.76,1.04)	0.142
Respiratory rate ≥40 per min	54.1 (297)	51.4 (302)	1.13 (0.95,1.34)	0.168
All signs of severity‡	42.3 (36)	30.9 (31)	0.81 (0.58,1.15)	0.242
Discharge	95.9 (292)	94.7 (296)	1.04 (0.94,1.15)	0.468

*Adjusted for baseline age, sex, height for age, weight for age, and weight for height z scores and pneumonia severity.

†Number of participants with the symptom at baseline.

‡Oxygen saturation <90%, lower chest wall indrawing, nasal flaring.

**Figure 4:**
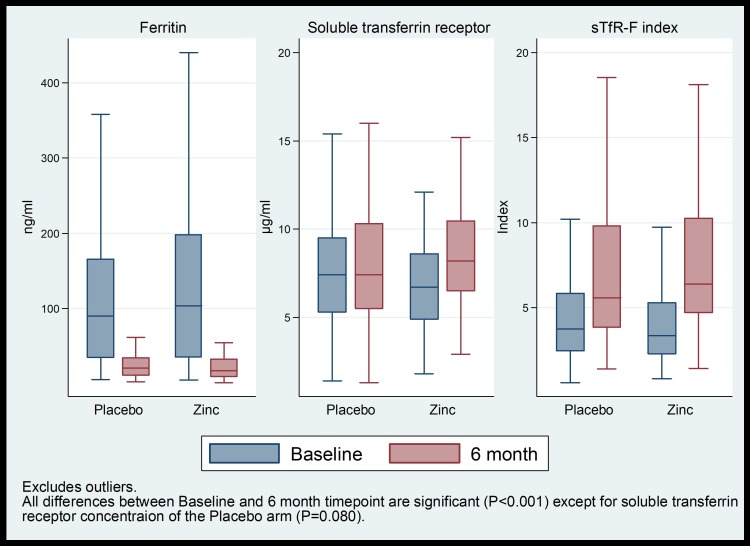
Plasma ferritin and soluble tranferrin receptor concentrations, and sTfR-F index at baseline and after six months of supplementation.

In the 6-month zinc supplementation sub-group there was no significant difference in the rate of height gain, HAZ, WHZ, mid upper arm circumference, anergy, and concentrations of plasma zinc, ferritin, sTfR, CRP, and in sTfR-F index (**Table 3**). Compared to baseline concentrations: median zinc concentration increased significantly and median ferritin concentration decreased significantly in both arms after six months; median sTfR concentration did not change in placebo arm but increased significantly in the zinc arm, and median sTfR-F index increased significantly in both arms (**Figure 4**).

**Table 3:** Treatment outcomes from six months of either placebo or zinc*

Outcomes	Placebo mean (N)	Zinc mean (N)	Difference (95% CI)*	*P*-value
Height (cm)	80.87 (92)	79.90 (89)	0.20 (-0.74,1.13)	0.681
HAZ	-1.01 (92)	-0.80 (89)	0.05 (-0.15,0.25)	0.611
WHZ	-0.50 (90)	-0.64 (88)	-0.20 (-0.46,0.05)	0.121
Growth rate (cm per month)	0.81 (98)	0.87 (100)	0.02 (-0.07,0.11)	0.704
Plasma zinc conc. (µmol/L)	19.92 (86)	22.04 (75)	2.53 (-0.75,5.81)	0.131
Plasma ferritin conc. (ng/ml)	57.79 (86)	26.07 (82)	-20.56 (-52.77,11.66)	0.211
Plasma soluble transferrin receptor conc. (µg/ml)	8.78 (88)	8.87 (87)	0.22 (-1.12,1.57)	0.746
Plasma C-Reactive Protein (mg/L)	15.57 (87)	8.44 (81)	-6.18 (-16.45,4.09)	0.238
Soluble transferrin-receptor-ferritin index (sTfR-F index)	7.69 (86)	9.02 (82)	1.57 (-0.68,3.83)	0.171
Outcomes	**Placebo n/N (%)**	**Zinc n/N (%)**	**OR (95% CI)**	***P*-value**
% with HAZ<-2	17/92 (18.5)	12/89 (13.5)	0.69 (0.31,1.54)	0.361
% with MUAC<12.5 cm	2/93 (2.2)	5/93 (5.4)	2.59 (0.49,13.68)	0.264
% Anergy	84/85 (98.8)	86/87 (98.9)	1.02 (0.06,16.64)	0.987

CI – confidence interval, OR – odds ratio, HAZ – height for age z score, WHZ – weight for height z score, sTfR – soluble transferrin receptor, MUAC – mid upper arm circumference

*Adjusted for baseline age, sex, HAZ, WHZ, pneumonia severity and zinc concentration.

## Discussion

This study did not show a reduction in treatment failure in children with severe pneumonia who were given daily zinc supplementation along with standard antibiotic treatment. There was no overall benefit in recovery from all signs of severe pneumonia. There was marginal benefit for time to resolution of LCWI and sternal retraction but this was relatively small and in the context of the study’s overall findings was of doubtful clinical significance.

Until now, efforts to identify a marker of zinc status have focused predominantly on the response to changes in dietary zinc intake [18]. The distribution of plasma zinc concentrations among a representative sample of a population has also been recommended to assess the risk of zinc deficiency in that population [19]. To our knowledge, this is the first randomised trial of the impact of zinc supplementation on severe pneumonia where zinc status has also been assessed within the trial by simultaneously measuring the response to zinc supplementation on plasma zinc, growth and immunity. Our population was not zinc deficient, as shown by absence of an impact of zinc supplementation on growth and immune competence, and the lack of efficacy of zinc supplementation may be due to an absence of deficiency. Even at baseline when plasma zinc concentrations would have been depressed by the high levels of inflammation only 33% of subjects had concentrations below the referent cut-off of 65 mg/L (9.9 µmol/L) and by the end of supplementation only 12% of the placebo and 6% of the zinc arms had low plasma zinc with median plasma CRP concentrations of 3.3 and 3.1 mg/L respectively. International Zinc Nutrition Consultative Group recommends that if more than 20% of the population has a serum zinc concentration below 9.9 µmol/L, the whole population should be considered to be at risk of zinc deficiency [20]. This suggests that our target population was not zinc deficient, which is also supported by the fact that only a small proportion of children were stunted.

In addition to poor dietary zinc intake [9], Gambian children are also at increased risk of zinc deficiency because of excessive endogenous loss of zinc due to environmental enteropathy and repeated diarrhoea episodes. In spite of these risk factors, our study children were not manifestly zinc deficient, though many were under-nourished. Rural African children are generally at risk of dietary zinc deficiency. However, the growth response to long term supplementation trials varied widely in trials conducted in Africa. An earlier study in Gambia among apparently healthy rural pre-school children did not show an impact of long-term zinc supplementation on growth [21]. Similarly, long-term trials of zinc supplementation among apparently healthy school children in rural Zimbabwe and in low income suburb in Uganda did not show overall growth response [22,23]. In contrast, Umeta et al showed significant effect of zinc supplementation on linear and ponderal growth in apparently healthy breastfed stunted and non-stunted infants in rural Ethiopia [16]. However, a recent meta-analysis that included 50 studies showed a small increase in height and weight [24]. In addition to subclinical zinc deficiency, there could be a deficiency of other growth limiting type 2 nutrients (as for example dietary protein) [25] in countries like Gambia, Uganda or Zimbabwe which were not given with zinc supplementation.

A subclinical zinc deficiency cannot be excluded. It is possible that repletion of zinc in sub-clinically zinc-deficient children does not confer benefit in recovery from severe pneumonia. Irrespective of whether our population was zinc-replete or had a subclinical deficiency the result does not suggest that zinc has any important pharmacological impact (ie, beyond the correction of deficiency) on the recovery of severe pneumonia in this population.

A majority of studies conducted in resource poor settings in Asia, Africa, and Latin America have not shown any benefit of zinc supplementation when used as an adjunct therapy in the management of severe pneumonia [26-29]. Only three studies showed overall benefit of zinc as an adjunct treatment of severe pneumonia [8,30,31]. Srinivasan et al [30] could not show any benefit for time to normalization of respiratory rate, temperature and oxygen saturation, although the study showed a significant reduction in case-fatality. However, in a stratified analysis, the reduction in mortality was limited to HIV infected children. A recent meta-analysis of nine zinc supplementation trials, as an adjunct treatment of severe pneumonia, did not show any benefit in time to recovery, length of hospital stay, treatment failure or change of antibiotics [32]. It is also possible that the low specificity of the WHO definition of severe pneumonia may have contributed to the study’s negative result as a lack of specificity in the diagnostic criteria for measuring study outcomes biases the risk/rate ratio towards null values.

General increase in plasma zinc in both arms of our study compared to baseline concentrations was likely due to plasma zinc being redistributed to tissues by an acute phase reaction during the episode of severe pneumonia and returning to normal plasma concentrations following recovery and the additional increase in the zinc concentration in the zinc arm being due to zinc supplementation. Lower plasma ferritin in both arms at six months compared to baseline can be explained by the normalization of elevated ferritin concentrations associated with the initial infection. Age was not correlated with ferritin at either enrolment (r = 0.03, *P* = 0.653) or follow-up (r = 0.08, *P* = 0.292). The similar ferritin concentrations in both arms and the median concentration of more than the WHO reference value ferritin (≥12 ng/mL) [33] at the six month time point suggest that there was no negative impact of long-term supplementation of zinc on iron status as judged by plasma ferritin. A recent study in children showed that zinc supplementation did not affect indicators of iron deficiency including plasma ferritin and transferrin receptor [34].

About a third of our study children were wasted (WHZ<-2) (20 in zinc and 25 in placebo arms). Zinc supplementation could have an impact in these children, but there was insufficient statistical power for detecting any difference in outcome in this sub-group. Further studies are required including only wasted children presenting with severe pneumonia.

The timeframe for availability of full laboratory results and the dampening effect of negative publication bias contributed to a delay in the publication of these findings.

## CONCLUSIONS

Overall, in this apparently non-zinc deficient population, zinc given as an adjunct treatment for severe pneumonia showed no benefit in treatment failure rates and showed marginal benefit in rapidity of resolution of some signs of severity, although this was not demonstrated across all signs of severity. This finding does not support routine use of zinc as an adjunct treatment in severe pneumonia in The Gambia and in other countries in similar settings.
